# External support and personal agency - young persons’ reports on recovery after family-based inpatient treatment for anorexia nervosa: a qualitative descriptive study

**DOI:** 10.1186/s40337-020-00293-5

**Published:** 2020-05-04

**Authors:** Jan-Vegard Nilsen, Trine Wiig Hage, Øyvind Rø, Inger Halvorsen, Hanne Weie Oddli

**Affiliations:** 1 Department of Psychology, University of Oslo, Oslo, Norway; 2 Regional Department for Eating Disorders, Division of Mental Health and Addiction, Oslo University Hospital, Oslo, Norway; 3 Institute of Clinical Medicine, University of Oslo, Oslo, Norway

**Keywords:** Eating disorders, Anorexia nervosa, Recovery, Adolescent, Qualitative research, User perspectives

## Abstract

**Background:**

Recommended treatment for adolescent anorexia nervosa (AN) is usually family-based and an overarching treatment aim is to empower the parents to manage the difficult meals and aid their child toward recovery. While family-based treatment prioritize collaborating with the parents, understanding the young persons’ views on recovery is also important. Understanding the young person’s views and ideas is relevant as this may facilitate the therapeutic alliance and thus aid the therapeutic process. The purpose of the present study was to investigate the reflections of young persons with a lived experience of anorexia nervosa, and what factors they consider important for the recovery process. All participants had been provided with a family-based inpatient treatment program, a program inspired by the core features of outpatient family-based treatment.

**Methods:**

Participants (*n* = 37) presented with an extensive treatment history, including outpatient and inpatient treatment for AN. Interview transcripts were analyzed by applying a predominantly inductive thematic approach to generate themes across participants.

**Results:**

The qualitative analysis generated a thematic structure entailing three levels. The superordinate theme, “Recovery is a long and winding journey: recognizing the need for support and highlighting the need for action”, captured three main themes, “Realizing you have a problem”, “Being involved in important relationships”, and “Giving treatment a real chance”.

**Conclusions:**

Our results demonstrated that although young persons with a lived experience of anorexia nervosa recognized the importance of support from others, they placed a distinctive emphasis on self-responsibility and determination. We recommend clinicians working within the recommended family-based treatment frameworks be curious about young patient’s subjective perspectives of the recovery process, as connecting with their views can potentially strengthen therapeutic relationships and facilitate change.

**Plain English summary:**

Recommended treatment for adolescent anorexia nervosa is usually family-based. These recommendations are supported by decades of research. In family-based treatment the overarching aim is to empower the young person’s parents to manage and take charge of the difficult situation caused by the eating disorder. As recommended family-based treatments usually prioritize collaborating with the parents, it is important to be curious on the adolescents own views of what is regarded as important for the recovery process. The present study offers insights into factors considered important to the recovery process by young persons with lived experience of AN. Although voicing the importance of enlisting support from families, friends, and loved ones, the young participants distinctly emphasized their own responsibility, motivation and self-determination as critical factors for recovery. Inspired by our findings, we recommend that clinicians address the young patient’s own preferred ideas for recovery during treatment.

## Background

Recovery from anorexia nervosa (AN) is not universally defined in the literature [1], and quantitative research has demonstrated that recovery rates vary exceedingly depending on the definition used [2, 3]. Moreover, recovery can be approached from several positions, as treatment providers, researchers and people with a lived experience may support different definitions. An alternative to the prevailing symptom-oriented recovery emphasis is recovery perceived from the position of people with a lived experience, emphasizing personal opinion and subjective meaning making [4].

Regardless of how one defines recovery from AN [5, 6], an interest in understanding what young persons with a lived experience perceive as important ingredients in the recovery process is important. Connecting with the patients’ own beliefs, values and preferences is considered essential for the design and delivery of evidence-based practice for eating disorders [7]. Using their clinical expertise, clinicians working with adolescents and families need to continually and wisely balance the best available research evidence and the treatment preferences of the patient and their family [8].

Research investigating patient’s or former patient’s perspectives on recovery has usually addressed this by asking adults or young adults to share their views [4]. This research has generally demonstrated that the journey toward restoring health could best be viewed as an intricate interplay between multiple factors [4, 9–11]. The importance of the person’s own willpower, motivation and agency on the one hand, and the significance of meaningful and supportive relationships on the other, has been highlighted in several studies [9, 12–15]. Together with these individual and interpersonal features, mastering daily life in general (such as coping with education, work, and being engaged in other meaningful activities) has been underlined as a crucial requirement for recovery [9, 12, 16]. Another recurrent theme has been the importance of treatment in general, and the significance of being actively involved to achieve progress [4]. In order to experience improvement, it seems the person has to develop ways to truly distance oneself from the eating disorder, both by actively taking charge of the recovery process (i.e., striving for a different ideal), and ultimately attaining a different identity in order to become fully recovered [9, 10, 13, 17]. Overall, qualitative findings shed light on the complex interplay between individual, relational and contextual factors when the journey toward recovery is perceived from the patients’ perspectives [4, 18–21]. Research investigating patient perspectives on recovery from the perspective of the young patient, literature is more limited [22]. In a review from 2015 that aimed to explore and synthesize the process of recovery from AN, the authors included only one study that involved young people (i.e., under 18) [4]. Even in this qualitative study over half of the participants were adults [17].

When a young person is suffering from AN, a family-based treatment approach is usually recommended [23]. Family therapy and family-based treatments have a long history in the treatment of adolescent AN [24, 25]. One possible consequence of emphasizing the family and parental role in obtaining recovery is less clinician investment in working directly with the young person afflicted with the ED [20, 26]. In manualized family-based treatment for AN the parental emphasis is especially clear, as the overall therapeutic aim in the critical first phase of treatment is to charge the parents with the responsibility for re-feeding and weight restoration. Consequently, the main therapeutic task becomes to empower the parents to manage this responsibility [27]. In such a family-based framework, enhancing the young person’s intrinsic motivation, promoting the adolescent’s responsibility for change, and working with adolescent-related issues, both within and outside the family, is usually toned down or postponed to the end of treatment [27, 28]. Although a predominantly family-based treatment approach is frequently portrayed in the literature as supported by promising research evidence [3, 29], researchers have started to question the evidence-base [30–32], describing its outcome in clinical trials as modest at best [3, 32] with some arguing that despite its promise, treatment needs to be augmented and better tailored to improve outcome [33, 34].

One way of augmenting the family-based treatment approach for adolescent AN is seen in the ongoing effort of enabling an enhanced family therapeutic focus at higher levels of care [35]. Although situated in various local treatment contexts, common features for these efforts is the overarching goal of aligning the intensified treatment (i.e., day-, residential- and inpatient treatment) with the core features associated with outpatient family based treatment [36–38]. Although such adaptations should be investigated further, preliminary outcome research show that this can be a promising way of providing treatment at higher levels of care for those who fail to respond to outpatient treatment [36, 37, 39].

Understanding better how young persons’ with lived experiences reflect upon important factors for recovery can provide additional knowledge, and help ascertain whether patient preferences and views align with the recommended treatment focus [20, 28]. Although the intricate relationship between the therapeutic alliance and ED outcome is not clearly understood [40] we do believe that managing a balance between treatment recommendations and the young person’s preferences is vital, as discrepancies can challenge therapeutic relationships and enhance conflicts. There is a paucity of research investigating the young person’s beliefs about what is considered important for recovery. As such, the present study can contribute with knowledge relevant for the ongoing effort of augmenting practices to tailor treatment to those failing to respond to the recommended first-line treatments for adolescent AN [34].

Research that focuses on the perspectives of young persons with lived experience with AN can provide important knowledge about how to improve and better tailor family-based treatment. With the present study, we aimed to investigate the perspectives of young persons with a lived experience of AN on factors related to the recovery process. By being situated within a higher level of care setting highly influenced by a family-therapeutic treatment approach, the present study can bring forth facets of recovery from a specific treatment context not included in previous research. The research question was, “what do adolescents with a lived experience of anorexia nervosa, who have taken part in a family-based inpatient treatment program at a specialized eating disorder unit, report as important factors for recovery?”

## Methods

### Context

This qualitative descriptive study formed part of a larger research project which aimed to investigate naturalistic ED outcome of family-based inpatient treatment for AN [36], treatment satisfaction [41], and the experiences of family members following family-based inpatient treatment [42]. Thirty-seven (64%) of 58 former inpatients (33 females/4 males), provided written consent to take part in this sub-study. For the sole participant under the age of 16 at follow up (i.e., age of consent), parental consent was also obtained.

### Treatment setting

During the family-based inpatient treatment program, up to five families were admitted at a time. The overarching treatment focus for the majority of participants corresponded to the first phase in outpatient FBT [27]. This meant that throughout the admissions, staff emphasized collaboration with parents, while the therapeutic focus on the young patient was more of an indirect one. Without aiming to strictly adhere to manualized FBT, the guiding treatment principles during admissions were inspired by outpatient FBT [27, 36]. The main therapeutic content consisted of conjoint and separated family therapy together with parental counseling, supplementary individual therapy and milieu therapy with the overarching aim of supporting parents to support their child during the stay. During the inpatient treatment program, parents were supported to manage meals and weight restoration, while staff aimed to externalize the ED and adhere to a non-blaming and non-etiological stance. Each young patient and family was allocated a multidisciplinary team. The nucleus of this team consisted of a child- and adolescent psychiatrist working closely with a clinical psychologist, and two or three nurses. Families were offered family therapy sessions at least twice a week. Some patients were offered supportive individual therapy in addition to family therapy. Nursing staff had daily scheduled conversations with both parents and the young person, for preparing meals and evaluating the ongoing process. Patients and parents took part in the weekly treatment meetings. At discharge, all patients and families were referred back to their local clinic for further outpatient treatment.

### Participants, recruitment and data collection

All participants (*n* = 37) had been admitted for family-based inpatient treatment between 2008 and 2014 and all had a primary admission diagnosis of AN. They presented with an extensive treatment history, including both outpatient and inpatient treatment prior to the family-based admission. Duration of ED prior to the family-based admission was on average 2.7 years (range; 0.5–6.0, *SD* = 1.8). Mean age at admission was 15.8 years (range; 12.4–19.5, *SD =* 1.8). The majority (33/37) were admitted voluntarily. Mean length of stay was 20.8 weeks (range; 3–58, *SD =* 13.5), including planned leaves from the ward as part of the treatment program. All families agreed to stay at the hospital with their child during the hospitalization. At the time of the follow up interview in 2015, the majority (65%) of the total sample (*n* = 37) had achieved normal body weight (i.e., estimated as achieving a BMI ≥18.5). Twenty two (59%) participants did not meet the criteria for any DSM-5 ED-diagnosis, 8 met criteria for AN, 2 for BN and 5 for OSFED. The mean age at the follow up interview was 20.2 years (range 15.8–25.3, *SD =* 2.6). The mean time period from discharge to the follow-up interview was 4.5 years (range; 1.3–7.0, *SD =* 1.7).

Ethics approval for this study was obtained from the Regional Committee for Medical Research ethics, South East Norway [REK2014/2223]. The 37 semi-structured interviews were administered by a team consisting of a senior researcher, two clinical psychologists, one psychiatrist and a psychiatric nurse. Twenty-six of the interviews were conducted on-site at the hospital, seven at the participant’s home, three by telephone, and one in-person elsewhere. All interviews (including telephone interviews) were audiotaped and transcribed verbatim by a research assistant and the first author. The qualitative interviews lasted between 30 and 100 min.

### Interview guide

The semi-structured interview guide was developed by a group of experienced clinicians to address a broad range of post family-based inpatient treatment user experiences. The guide was not constructed based on a specific theoretical model. The interview guide was structured into three sections, including questions covering the pre-admission phase, the admission and post-admission phase. Most relevant for the present paper’s analysis was the post-admission items, and particularly the following questions: “Looking back on your life and the changes that have happened related to your eating disorder – how would you describe important turning points?” and “What do you think is most important in recovering from an eating disorder?”

### Qualitative data analysis

To provide an overall structure for the analysis, we applied a thematic analysis (TA) guiding framework [43]. TA is commonly recognized as a pragmatic and flexible framework entailing six steps to guide the researchers: 1) familiarizing yourself with the data, 2) generating initial codes, 3) searching for themes, 4) reviewing themes, 5) defining and naming themes, and 6) producing the report [43]. To enable as much diversity as possible, we decided to include all eligible patients (*n = 37*) in the analysis.

To manage the quite large number of transcripts, the QSR International’s Nvivo11 Software [44] was used for both the initial phase of sentence by sentence coding (Step 2) and in aiding the iterative process of going back and forth between the gradually developing thematic map and checking back with the raw data in reviewing and ensuring that the evolving thematic map provided a good fit with the raw data (Steps 4 and 5).

Together with the first author reading and re-reading the complete data set several times, all authors familiarized themselves with reading selected parts of the data material (Step 1). The first author had the overall lead in initial coding, interpreting and moving the process of theme development forward, toward finalizing the analysis and writing up the first draft (i.e., Steps 2–6 in thematic analysis). Although we did not adhere to a strict schedule of co-analyzing the transcripts, scientific rigor and trustworthiness [45, 46] were ensured by the research team doing parts of the analysis together. This co-constructive effort was secured by TWH reviewing and supervising the gradual steps initiated by the first author, and HWO supervising the process of analyzing the transcripts as a whole. During the analysis both TWH and HWO performed the role as a “critical friend” [47]. Reflexivity was thus continually addressed through frequent dialogues and team meetings where “the two friends” together with the first author critically questioned the emerging theme development, and encouraged different interpretations from different positions [48, 49].

**Fig. 1:**
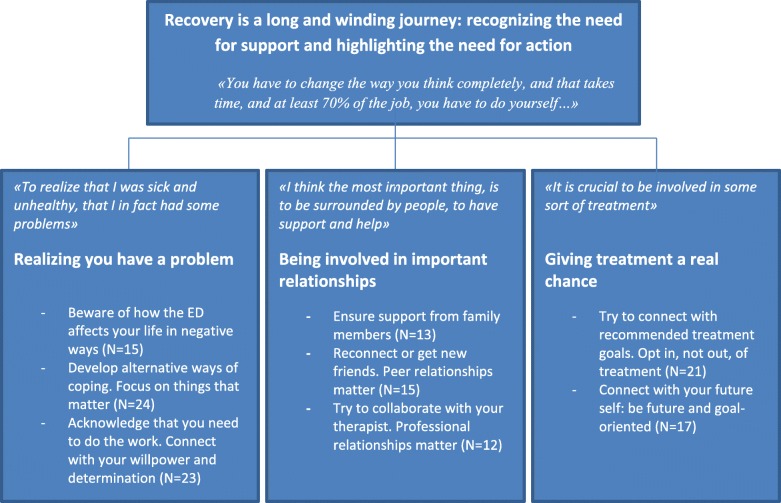
represents the thematic structure on altogether three levels: superordinate theme, capturing 3 main themes with adjacent subthemes. Numbers in parenthesis equals the number of participants sharing views within each subtheme

Overall, the analysis was predominantly inductive and hence not driven by a specific theoretical approach. The iterative process of developing, reviewing and finally defining and naming themes (Steps 3 to 5) was informed by a combination of both a semantic and interpretative stance. Semantic in this context meant that we initially aimed to navigate our curiosity predominantly to the surface level [43]. As the analysis proceeded, we recognized that a more interpretative lens was necessary to allow more nuance and richness to the analysis. Reviewing the process, we recognized that Steps 1 to 3 were mainly influenced by a semantic level of analysis, with Steps 4 and 5 integrating more interpretation. As is common in qualitative analysis, the finalized thematic structure underwent several major and subtle corrections before we finalized the thematic map which best represented and communicated the views of the participants. To provide readers with transparency about the distribution of accounts across themes and an opportunity to evaluate robustness of findings, we added numbers to the subthemes [50] (see Fig. 1 for details).

## Results

The qualitative analysis generated a thematic structure entailing three levels (see Fig. 1 for details). Illustrative quotes are provided on both the main theme and subtheme level, together with brief illustrations on the superordinate and main theme levels in Fig. 1. All names provided are pseudonyms.

### Superordinate theme: recovery is a long and winding journey: recognizing the need for support and highlighting the need for action

The superordinate theme represents an abstraction of the three main themes and their adjacent subthemes, including a) participants predominantly viewed recovery as a gradually evolving process which typically included episodes of progress and setbacks and b) although the majority of the participants viewed support from others and their own agency as important ingredients for getting better, the latter was particularly emphasized. Furthermore, the superordinate theme captured the advantages of viewing the ED as a problem, or as problematic, in order to mobilize efforts toward change.

### Main theme 1: realizing you have a problem

This main theme captured the participants’ views on the necessity of recognizing that the ED represents a real-life problem, and that you yourself need to fight to achieve change, as problems do not just pass with time.

To realize or admit that I was ill, like, that I really had some problems, that, *that* I feel was quite important. And I thought, yes, I started to realize that I had to do some things myself. I kind of had to decide that for myself, to *do* something. That’s important [Sarah, 19]

#### Subtheme 1: *Beware of how the ED affects your life in negative ways (N = 15)*

Although some of the participants noted past and present ambivalence toward recovery, several accentuated the importance of being aware of how the ED affects relationships negatively and obstructs desired goals and future dreams. Reflecting back, all but one participant reflected on a slowly evolving realization of how the ED affects your life in negative ways.

It’s important to realize and see more clearly the negative influences the ED has, because it is, after all, a way of handling difficulties or mastering life, right, so if what you get from the ED is more or better than the burden you experience, then it’s difficult, to let go. But if what you get from the ED is shit, and in fact worse than the other struggles you’ve got, then it becomes easier. But it is difficult. It’s not easy to be attentive to the negative consequences the ED will have [Polly, 23]

#### Subtheme 2: *Develop alternative ways of coping. Focus on things that matter (N = 24)*

Only three participants explicitly conceptualized the ED as a “coping strategy” during their interviews. However, this subtheme captured our understanding of the participants’ tendencies to conceive the ED or ED-behaviors as representing different ways of coping with other difficulties as low self-esteem, difficult life experiences or relationships, or as a means of regulating emotions. Several of the participants emphasized the necessity of letting go of the ED or ED behaviors in order to recover, and their reflections suggested that focusing on things that really matter can aid this process.

It’s been crucial to accomplish high school, and to get a driver’s license, to start with higher education. There are new goals all the way, and it feels really great to accomplish those, and, it’s this sense of mastering, which is very important. To feel you can live a pretty normal life, where the focus is on everything else *but* body and food. That’s something I’ve been working with, to shift the focus [Andrea, 20]

#### Subtheme 3: *Acknowledge that you need to do the work. Connect with your willpower and determination (N = 23)*

The majority of the participants were clear that they viewed their own willpower and decision-making as necessary ingredients in the recovery process. Several challenged the idea of the existence of any ideal or perfect moment for change, and rather urged fellow peers to start to work actively for change, now.

You’re never ready for it. You’ll never wake up one morning and suddenly think; now I am ready! Because, if this was how it was, it would have happened. So it’s something you need to *do*, that’s how it is. Just start! Just start. Make a habit out of it, and, easier said than done, but really. Never wait for the perfect moment. That’s not going to happen. No, you will never be fully ready [Polly, 23]

### Main theme 2: being involved in important relationships

This main theme captured views which emphasized the importance of others. Although recognizing support from parents, peers and others as important for the recovery process, the majority of the participants made a point that they themselves must reach out and do what they can to be involved in important relationships. Quite a few of the participants also emphasized the potential of a collaborative and supportive relationship with health care professionals. A few shared that being in love and engaged in a romantic relationship helped shift the focus towards more important aspects of life and thus, minimized the influence of the ED. Even relationships with pets were seen as potentially aiding toward recovery by some. As Joanne viewed it, relationships can be both supportive per se, and also represent a stepping stone towards accommodating other meaningful aspects of life.

Most important is to have people around, support and help and, yes, you need to understand that there are better things than just thinking of food, and of course you need to want it [change] yourself, but that usually progresses out of relationships so … [Joanne, 21]

#### Subtheme 1: *Ensure support from family members (N = 13)*

Many of the participants viewed support from parents and siblings as important for getting better. Quite a few were clear that parental support and parental involvement in treatment had been very important for getting better. Having family members who behaved in ways that enhanced the feeling of being understood seemed crucial, as the opposite could risk the likelihood of enhancing both feelings of loneliness and opposition. Reflecting on support from family members, several of the participants also stressed the importance of opening up and actively welcoming the support, as opposed to avoiding or opposing family-members’ engagement and involvement.

The fact is that people around you want the best for you, they want to help you and you really have to understand that they want to support you, and that they’re not your enemies that want to hurt you. That’s the EDs intention; it wants me to believe that everybody is cruel and want to hurt me [Kate, 21]

#### Subtheme 2: *Reconnect or get new friends. Peer relationships matter (N = 15)*

Although quite a few emphasized the importance of support from parents and family members during the recovery process, several of the participants underscored the importance of peer relationships. Specifically, the importance of keeping in touch with friends during treatment and illness was emphasized, as well as actively striving to reconnect if friendships had halted. Quite a few encouraged young persons to develop new friendships if feeling alone, reminding others that friends do not just show up; you need to take social initiatives yourself.

I worked really hard to get back my friends. I remember I had to, in the beginning. I had to invite myself to all parties. I remember thinking this was embarrassing and really humiliating, but still I thought that I really had to do it, to give them the chance to know me over again, and take me into their lives, and that worked out really well. Now I have several friends, and I don’t need to invite myself any longer, I’ve become a part of them [Brenda, 22]

#### Subtheme 3: *Try to collaborate with your therapist. Professional relationships matter (N = 12)*

Although mixed experiences were voiced when reflecting upon past therapeutic encounters, more than a few of the participants emphasized that being engaged in therapy and therapeutic collaborations can be vital for change to happen. Here too, several used the opportunity to reflect upon the importance of becoming actively engaged in the relationship with the health care professionals, alluding that little or nothing will happen if the young person remains silent or too passive or ultimately opposes the therapist.

I now feel that I’ve met the person I can manage to get well together with. My key worker is so secure and I’ve managed to do a lot of important work and progress together with her [Anna, 18]

### Main theme 3: giving treatment a real chance

This main theme captured the participants’ views on treatment as a potentially active ingredient for the process of recovery. The theme captured participants’ views about the importance of actively aligning with recommended treatment goals (i.e., normalizing eating behaviors and attaining normal weight) and the importance of working through treatment ambivalence and resisting the temptation to opt out of treatment. Additionally, a potential domain for therapy was accentuated through their reflections on goal attainment (i.e., Subtheme 2).

Dare to let go, and give treatment a chance [John, 22].

#### Subtheme 1: Try to connect with recommended treatment goals. Opt in, not out, of treatment (N = 21)

I haven’t thought much about having kids. Still, I think it is important to stay in treatment, because I want to be able to take good care of my kids, which is a huge motivation for me, actually … [Catherine, 20]

Over half of the participants emphasized the importance of being invested in some sort of treatment. It was as if several of the participants wanted to inspire others struggling with EDs to give treatment a real chance. Although being involved in treatment was not necessarily viewed as synonymous with achieving change, more than a few participants highlighted the significance of opting in and not out of treatment. Looking back, quite a few realized that they had wanted to invest even more in treatment encounters, if they could rewind and do things over. The majority of the participants emphasized the significance, and even the necessity of, striving for normalizing eating behaviors for letting go of the ED, while others stressed the importance of giving normal weight a chance.

You have to give normal weight a chance. Not just decide in advance that; “that’s not for me”, “*that* I don’t dare”, “that I don’t want”. It’s all about being bold enough to do the changes [Jane, 21]

#### Subtheme 2: Connect with your future self: be future and goal-oriented (N = 17)

Ask yourself, why, ehm, why do you do this? What do you want to get out of your life? What are your true dreams? What is your greatest wish? [Maria, 21]

Several of the participants noted that reaching new personal milestones had reinforced hope, motivation and self-respect. As a consequence, they indirectly supported the notion of the therapeutic benefit of clarifying attainable goals of personal significance. Several of the participants felt that having a future- and goal-oriented focus, both distant and proximal, would be beneficial to emphasize in treatment and fruitful for the young person with AN.

Try to find something in your everyday life that is positive for you and that you really have an urge to accomplish, and if you have a goal you really long for, go for it, because when you accomplish it, that joy! [Esther, 19]

## Discussion

This study aimed to investigate what adolescents with a lived experience of anorexia nervosa, who had taken part in a family-based inpatient treatment program at a specialized eating disorder unit, reported as important factors for recovery. As demonstrated by the superordinate theme, “Recovery is a long and winding journey: Recognizing the need for support and highlighting the need for action”, the results revealed that participants distinctively emphasized the importance of support from others as well as personal responsibility. Although support from parents, siblings, health care professionals, friends and romantic partners was valued, the centrality given to their own motivation and self-determination was especially striking in this study. A self-orientation stance was a central finding throughout the thematic analysis, as the main themes *realizing you have a problem*, *being involved in important relationships*, *giving treatment a real chance* all captured views contingent upon the individual.

The importance ascribed to the person’s own agent self is an aspect embedded in recovery stories documented previously in the literature [9, 13, 22]. Still, these views, emphasizing the young person’s own wishes (i.e., motivation), willpower and determination, are particularly interesting in the present context, as our treatment setting offered family-based treatment of AN, which prioritizes the parental role in treatment and postpones the adolescent’s role in treatment. Although the treatment offered did not strictly adhere to manualized outpatient FBT [27], the majority of the participants had experienced extensive efforts to involve family in treatment, including family-based inpatient treatment [36].

Family relationships are often significantly, and adversely, affected when a young person develops AN, and involvement of the young persons’ family in treatment is recommended by international treatment guidelines [23]. Supporting parents to support their loved one is an overarching and integral treatment priority for family-based treatment models [24, 27, 51]. The predominant role of parents is based upon the assumption that young individuals afflicted with the ED lack the ability to make rational and healthy treatment decisions due to inherent characteristics of the eating disorder (e.g., the ego-syntonic symptom quality, effects of malnutrition, ambivalence to change, treatment resistance). As a consequence, it becomes vital during treatment to prioritize the support of the less afflicted and legally responsible family members (i.e., the parents), and to provide them with the necessary skills and confidence to make health promoting choices on behalf of the young person. By default, the main aim of treatment is to provide sufficient support to ensure that parents are capable of taking charge of the refeeding process to restore weight and normalize eating patterns [28]. Although family-based treatments have a promising evidence-base [29], a large proportion of patients and families participating in clinical trials fail to achieve remission [25, 30, 31]. A more modest outcome becomes especially visible when strict remission criteria are applied [3]. Consequently, several questions remain on how we can optimize treatment to enable a better fit for both the young person and his and her family.

One question brought forth by our findings is whether adolescent AN treatment sufficiently enables a focus on the young person, and whether treatment succeeds in aligning with the young person’s own preferences and values, a hallmark of evidence-based practice [7]. In particular, it may prove relevant for individuals presenting with a clinical picture associated with non-response to FBT [25], or for individuals with extensive and not yet efficient treatment efforts, and finally, when the patient’s age or developmental stage demand greater focus on individuation and autonomy [26, 52].

Qualitative research has found that adolescents value many core aspects of family-based treatment, such as increased responsibility attained by parents and externalization of the ED [20]. Still, others have found that some adolescents view family-based approaches as neglecting vital individual aspects valued as important [15, 18, 20]. Although the present study does not argue against working within a predominantly family-based framework, it may be relevant to investigate further whether there are issues valued as important from the young person’s position that are insufficiently addressed in recommended ED treatments [20, 53]. Rather than challenging a family-based approach, these findings could be interpreted as shedding light on potential conflicts and dilemmas clinicians may encounter in providing family-based AN treatment, especially in the case of non-remission or relapse. The present study, in our view, suggests the importance of endorsing an increased adolescent-focused approach within a family-based framework, rather than advocating for a separate adolescent-focused therapy for the adolescent.

Reassuringly, the findings revealed that participants urged peers to opt in, and not out, of treatment, and that normal weight is considered as essential, and even prerequisite, for improvement. These findings align with previous qualitative research demonstrating the centrality of treatment for recovery [4, 18]. Findings showed that important relationships were perceived as beneficial for the recovery process. This is consistent with both theory and clinical observations illustrating that family dynamics are afflicted when a young person develops AN, and is in line with recommendations to involve the entire family in treatment [24, 54]. However, results also demonstrated that friends and romantic relationships, even pets, are viewed as important factors in recovery. This implies that clinicians and treatment providers should offer treatments that are attentive towards the young person’s wider social context, which concurs with prior studies [53, 55–57].

Rather than pinpointing specific turning points, the majority of participants in this study reflected that recovery was an emerging and gradual process to overcome their eating disorder. Few shared explicit examples on discrete turning points, which could also be due to study design or the relatively young age of the sample. Personal narratives on turning points may continue to evolve and become construed as persons become older [9].

Overall, research investigating adolescents’ “insider perspectives” on what is viewed as personally important for recovering from an ED is essential, as treatment outcome for this population is considered modest at best [6]. Consequently, many unanswered questions remain to be answered regarding how we can more efficiently provide and personalize treatment for adolescents needing specialized care for AN [58].

### Strengths and limitations

Investigating young persons’ views about factors important for recovery is an understudied area. This issue is worth investigating as it is important to understand whether young patients’ preferred ideas and views aligns with recommended family-based treatments focusing on parental responsibility. An important limitation is related to the interview guide and data collection. The semi-structured interview covered a wide range of questions assessing participants’ treatment experiences and was not developed with the sole aim of investigating the current study’s research question (i.e., young persons’ beliefs about important factors for recovery). More in-depth and nuanced reflections might have been obtained if the interview guide and interview process had been designed specifically for the sole purpose of this study. Four of the interviewers who collected data were previously employed at the treatment unit. As such, interviewees might have minimized disclosure of relevant information due to concerns of disappointing the interviewer. On the other hand, familiarity with the interviewer could also be viewed as strength, as participants might have felt at ease in disclosing sensitive information. Participants were not asked to provide feedback on transcripts or preliminary findings, which could have also provided greater depth and enhanced validity of the results.

## Conclusions

This study offers valuable insights into factors considered important to the recovery process by young persons with lived experience of AN. Although voicing the importance of enlisting support from families, friends, and loved ones, participants distinctly emphasized their own responsibility, motivation and self-determination as critical factors for recovery. The view that external support is important aligns with the predominant relational stance embedded in a family-based treatment approach for AN. Whereas the self-orientation stance (i.e., the importance the participants place on their own agency), suggests that increased therapeutic focus is needed to facilitate the young person’s own motivation and agency while working within a family-based framework, a framework that typically emphasizes fostering parental agency. Inspired by our findings, we recommend that clinicians address the young patient’s own preferred ideas for recovery during treatment. This stance aligns with an evidence-based practice framework and is oriented toward the young person’s own ideas and preferences, which may help foster treatment engagement and ultimately aid change.

## Abbreviations

AN: Anorexia nervosa
FBT: Family-based treatment
TA: Thematic analysis
